# Comparison of tumor growth assessment using GFP fluorescence and DiI labeling in a zebrafish xenograft model

**DOI:** 10.1080/15384047.2023.2234140

**Published:** 2023-07-16

**Authors:** Yaal Dryer, Joos Berghausen, Karen Creswell, Eric Glasgow, Tinatin I. Brelidze

**Affiliations:** a Department of Pharmacology and Physiology, Georgetown University Medical Center, Washington, DC, USA; b Department of Oncology, Georgetown University Medical Center, Washington, DC, USA

**Keywords:** Breast cancer, MDA-MB-231, xenograft, zebrafish, DiI, GFP, lipophilic dye

## Abstract

DiI is a lipophilic fluorescent dye frequently used to label and trace cells in cell cultures and xenograft models. However, DiI can also transfer from labeled to unlabeled cells, including host organism cells, and label dead cells obscuring interpretation of the results. These limitations of DiI labeling in xenograft models have not been thoroughly investigated. Here we labeled green fluorescent protein (GFP)-expressing MDA-MB-231 cells with DiI to directly compare tumor growth assessment in zebrafish xenografts using the DiI labeling and GFP fluorescence. Our results indicate that the DiI based assessment significantly overestimated tumor growth in zebrafish xenograft models compared to the GFP fluorescence based assessment. The imaging of DiI labeled GFP-expressing MDA-MB-231 cell cultures indicated that the DiI labeling of the membrane is uneven. Analysis of the DiI labeled GFP-expressing MDA-MB-231 cell cultures with flow cytometry indicated that the DiI labeling varied over time while the GFP fluorescence remained unchanged, suggesting that the GFP fluorescence is a more reliable signal for monitoring tumor progression than the DiI labeling. Taken together, our results demonstrate limitations of using DiI labeling for xenograft models and emphasize the need for validating the results based on DiI labeling with other orthogonal methods, such as the ones utilizing genetically encoded fluorophores.

## Introduction

Xenograft models are frequently used to study tumor progression.^1^ In xenograft studies, cancer cells are injected into animal models to examine tumor growth, metastasis, and response to therapeutic agents. While most of the xenograft assays are performed in mouse models, zebrafish recently emerged as a popular cost-effective alternative with high-throughput capabilities.^2–4^ The genetic similarity between humans and zebrafish is high, with approximately 70% of human genes and 80% of human disease-associated genes represented by at least one zebrafish orthologue.^5^ The transparency of zebrafish embryos affords an easy assessment of cancer cell growth, invasion, and extravasation, including at a single-cell level using a high-resolution imaging and immunofluorescence staining.^6–9^ The use of zebrafish model is also advantageous for patient derived xenografts (PDX), where patient-derived cancer cells are injected into animal models. Zebrafish embryos lack a mature immune system until 4–6 weeks post fertilization.^10^ This minimizes the rejection of patient-derived cancer cells and avoids the necessity of developing immunodeficient animals used for murine cancer models.^11^

In order to track cancer cells in xenograft models it is imperative to use stable cell labels. GFP and other genetically encoded fluorophores have been extensively used to monitor cancer progression.^12–15^ However, for this approach cancer cells have to be genetically modified to express the fluorescent proteins, which could be time consuming and not always possible. Another commonly used approach to trace cancer cells is by labeling them with lipophilic dyes. DiI is one of the most frequently used lipophilic dyes in cancer research, including studies using animal xenograft models and, more recently, PDXs.^3,7,16–18^ DiI partitions into cellular membranes due to its similarity to the membrane lipid structure.^19^ DiI is nontoxic to the labeled cells, does not alter cell proliferation and retains fluorescence over a long-period of time.^20–22^ However, using DiI for cell tracing has a number of limitations, including the transfer of DiI from labeled into unlabeled cells and retention of DiI in the cell membranes of dead cells that could lead to the overestimation of the number of labeled viable cells.^23–26^

Here we investigated the limitations of the DiI labeling approach for the assessment of tumor growth in zebrafish xenograft models using stably GFP-expressing MDA-MB-231 cells that were labeled with DiI. Our results indicate that the DiI labeling significantly overestimated tumor growth in comparison with the GFP fluorescence. The overestimation is likely due to the transfer of DiI from the labeled cancer cells into zebrafish tissue and retention of DiI labeling in dead cells. Imaging of the cultured GFP-expressing MDA-MB-231 cells indicated punctate labeling with DiI. Moreover, flow cytometry analysis of the cultured GFP-expressing MDA-MB-231 cells indicated that the DiI labeling varied over time, as opposed to the stable GFP fluorescence levels. Our results indicate that the tumor growth assessment based solely on DiI labeling could be inaccurate and needs to be corroborated with other methods.

## Results

### Xenograft tumor assessment using GFP fluorescence

**Figure 1.**
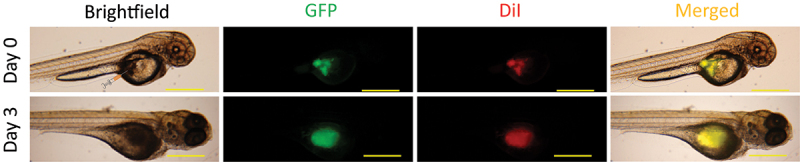
Schematic of zebrafish xenograft assay. Representative brightfield and fluorescent images of zebrafish injected with GFP-expressing DiI labeled MDA-MB-231 cells and their overlay on Day 0 and Day 3 after the injection. The cells were injected into the yolk sac of 2 dpf zebrafish, as illustrated on the brightfield image for Day 0. Scale bar = 500 μm.

**Figure 2.**
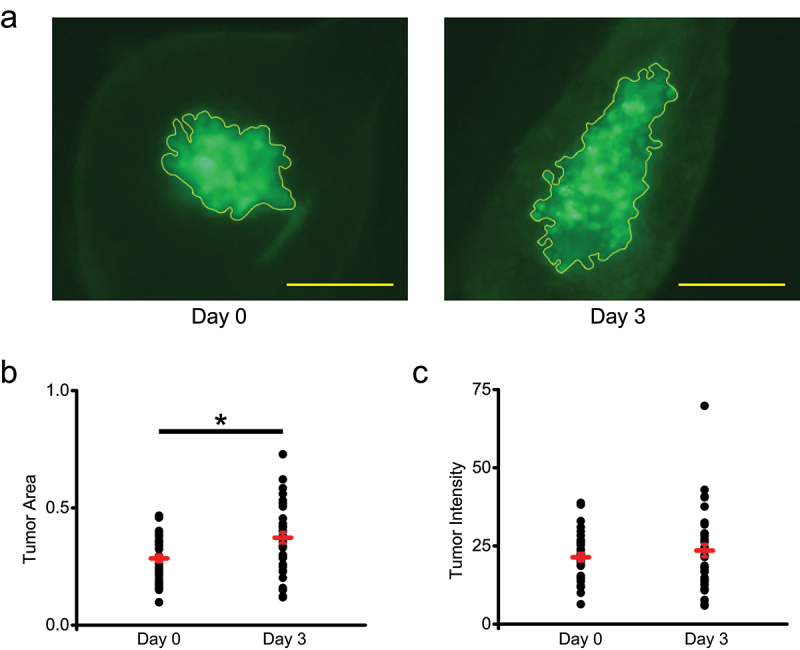
GFP fluorescence of zebrafish xenografts of GFP-expressing and DiI labeled MDA-MB-231 cells. (a) Representative image of a zebrafish xenograft tumor area outlined in yellow based on the GFP fluorescence on Day 0 and Day 3 post-injection into 2pdf zebrafish embryos. Scale bar = 50 μm. (b) Quantification of tumor area based on the GFP fluorescence on Day 0 and Day 3. Average tumor area was 0.28 ± 0.01 on Day 0 and 0.37 ± 0.02 on Day 3 (*n* = 37). (c) Quantification of tumor intensity based on the GFP fluorescence on Day 0 and Day 3. Average tumor intensity was 21.39 ± 1.25 on Day 0 and 23.52 ± 2.01 on Day 3 (*n* = 37). *P < .05, paired-samples Student’s T-test.

In order to compare the potential differences for assessing tumor growth using GFP fluorescence versus DiI labeling, we selected MDA-MB-231 cells commercially available from InnoProt that express GFP as a cytoplasmic protein. To allow for a direct comparison of GFP- and DiI-based assessments, the GFP expressing MDA-MB-231 cells were labeled with DiI and injected into zebrafish larvae at 2 days post-fertilization (dpf), as illustrated in Figure 1. Both tumor size and fluorescence intensity were determined immediately post-injection (Day 0) and 72 hours post-injection (Day 3). Tumor area selection for one of the xenografts using GFP fluorescence is illustrated in Figure 2a for Day 0 and Day 3. The quantitative assessment indicated a statistically significant increase in the average tumor area for the examined xenografts on Day 3 (Figure 2b, *P* value of $4\times 10^{-5}$ based on the paired-samples Student’s T-test). We also observed an increase in the tumor intensity on Day 3, however, it was not statistically significant (Figure 2c).

### Xenograft tumor assessment using DiI labeling

**Figure 3.**
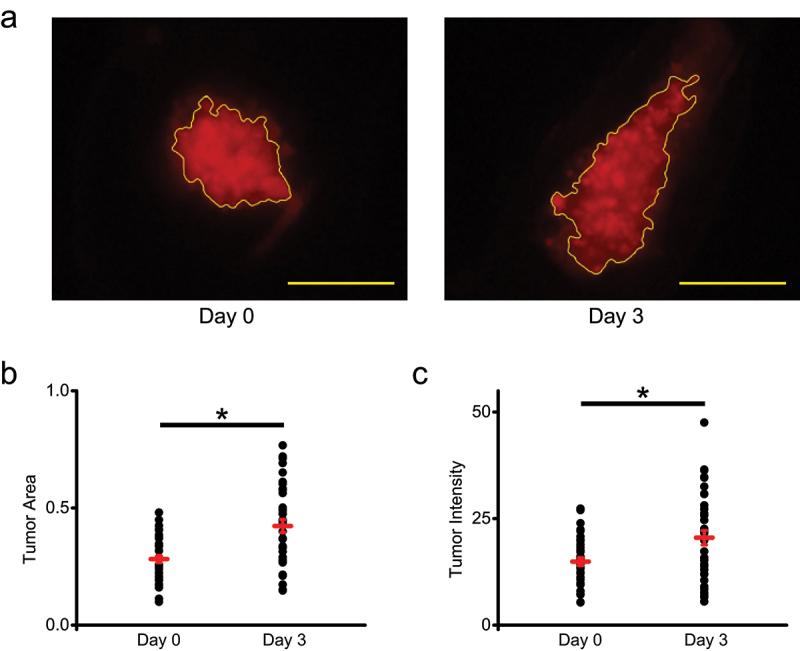
DiI labeling of zebrafish xenografts of GFP-expressing and DiI labeled MDA-MB-231 cells. (a) Representative image of a zebrafish xenograft tumor area outlined in yellow based on the DiI labeling on Day 0 and Day 3 post-injection into 2pdf zebrafish embryos. Scale bar = 50 μm. (b) Quantification of tumor area based on the DiI labeling on Day 0 and Day 3. Average tumor area was 0.28 ± 0.01 on Day 0 and 0.42 + 0.03 on Day 3 (*n* = 37). (c) Quantification of tumor intensity based on the DiI labeling on Day 0 and Day 3. Average tumor intensity was 14.91 + 0.94 on Day 0 and 20.51 + 1.71 on Day 3 (*n* = 37). *P < .05, paired-samples Student’s T-test.

To investigate potential differences in the tumor size determined based on the GFP fluorescence versus DiI labeling, we imaged the same xenografts as above using DiI fluorescence. Tumor area selection for the same xenograft as in Figure 2a using DiI fluorescence is illustrated in Figure 3a for Day 0 and Day 3. For Day 0, the average tumor area determined based on the DiI fluorescence was the same as the tumor area determined with the GFP fluorescence (Figures 2b and 3b), indicating that the GFP and DiI autofluorescence levels are comparable and should not affect the conclusions of our study. Further quantitative assessment of the DiI fluorescence indicated a statistically significant increase for both the average tumor area (Figure 3b, P value of $1\times 10^{-7}$ based on the paired-samples Student’s T-test) and intensity on Day 3 (Figure 3c, *P* value of $1\times 10^{-5}$ based on the paired-samples Student’s T-test). The amount of DiI in a given xenograft is expected to stay at the same level throughout the experiment, determined by the amount of DiI labeling in the cells injected on Day 0. Therefore, the statistically significant increase in the DiI fluorescence was unexpected.

### DiI labeling overestimates tumor growth in xenografts

**Figure 4.**
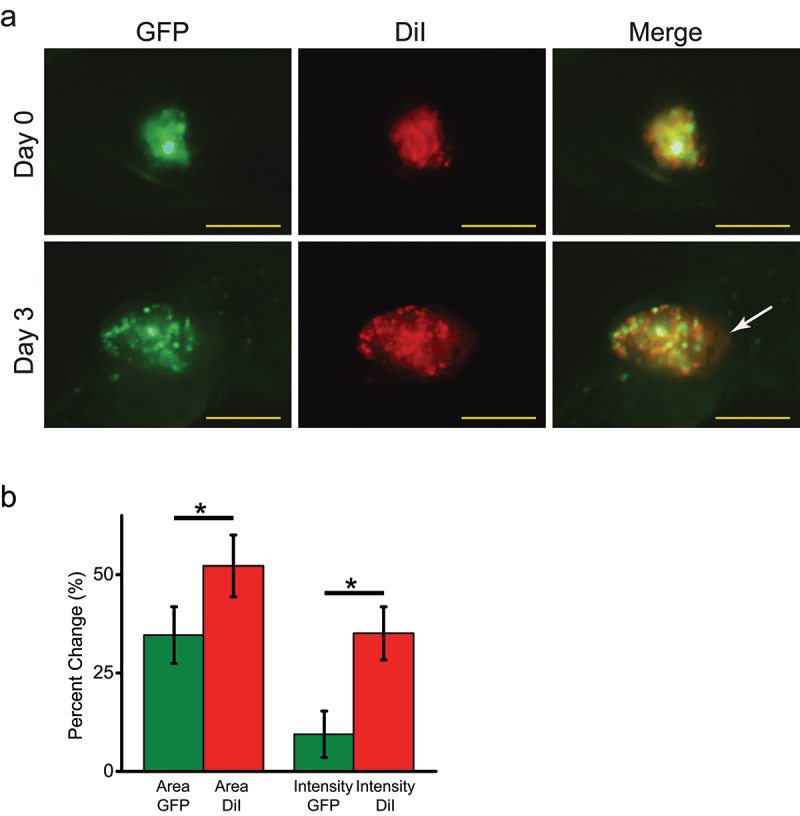
Comparison of GFP fluorescence and DiI labeling of zebrafish xenografts of GFP-expressing and DiI labeled MDA-MB-231 cells. (a) Representative DiI, GFP and merged fluorescence images of a zebrafish xenograft tumor on Day 0 and Day 3 post-injection into 2pdf zebrafish embryos, as indicated. The white arrow indicates the DiI labeled border area of the tumor that lacks GFP-fluorescence. Scale bar = 50 μm. (b) Percent change in the tumor area and intensity determined based on the GFP fluorescence or DiI labeling. Average percent change in tumor area was 34.62 ± 7.2 based on the GFP fluorescence and 52.22 ± 7.86 based on the DiI labeling (*n* = 37). Average percent change in tumor intensity was 9.45 ± 5.89 based on the GFP fluorescence and 35.08 ± 6.75 based on the DiI labeling (*n* = 37). *P < .05, paired-samples Student’s T-test.

Although the tumor area outlines based on the GFP fluorescence and DiI labeling were similar for the xenograft shown in Figures 2a and 3a, the GFP fluorescence and DiI labeling did not overlap for many of the other xenografts in our study. An example of such lack of overlap in GFP and DiI fluorescence is shown in Figure 4a, where a merged GFP and DiI fluorescence xenograft image for Day 3 shows the tumor border area, indicated with a white arrow, that lacks GFP fluorescence while still displaying DiI labeling, although of decreased intensity relative to the “central” areas of the tumor, suggesting potential transfer of the DiI labeling from the DiI labeled cancer cells into the host tissues. Consistent with this, the average percent change in the tumor area estimated based on the GFP fluorescence was significantly smaller than the one estimated based on the DiI labeling (Figure 4b; *P* value of 0.0028 based on the paired-samples Student’s T-test). The change in the tumor intensity estimated based on the GFP fluorescence was also significantly smaller than the one estimated based on the DiI labeling (Figure 4b; *P* value of $2\times 10^{-4}\;$ based on the paired-samples Student’s T-test). These results indicate that labeling xenografts with DiI leads to a statistically significant overestimation of tumor growth.

### Comparison of GFP fluorescence and DiI labeling for cultured cells

**Figure 5.**
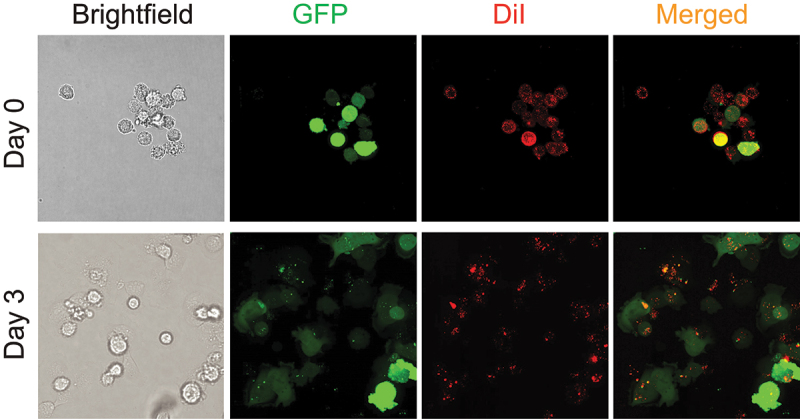
Imaging of GFP fluorescence and DiI labeling of MDA-MB-231 cell cultures. Representative brightfield and GFP, DiI and merged fluorescence images of the MDA-MB-231 cells on Day 0 and Day 3 of the experiment.

To gain further insight into the differences in the tumor growth assessment using GFP versus DiI labeling, we used imaging and flow cytometry to track the changes in the fluorescence signals for cultured cells as they divide. For the cell culture imaging, GFP expressing MDA-MB-231 cells were labeled with DiI. A fraction of the labeled cells was imaged immediately after labeling with a confocal microscope to assess the efficiency of DiI labeling. The labeled cells were also cultured and imaged on Day 3 of the experiment. Representative images of cells taken on Day 0 and Day 3 are shown in Figure 5. The initial labeling efficiency with DiI was high as most of the cells were labeled with DiI on Day 0. Since the MDA-MB-231 cells express GFP as an intracellular protein, the GFP fluorescence was evenly distributed inside the cells. Strikingly, the DiI labeling was punctate in appearance on Day 0 and Day 3, labeling limited spots of the cellular membranes. Moreover, the punctate appearance was more uneven on Day 3. Such an irregular labeling may prevent an even transfer of the dye to the daughter cells during the cell division, decreasing the number of DiI labeled cells with cell division.

**Figure 6.**
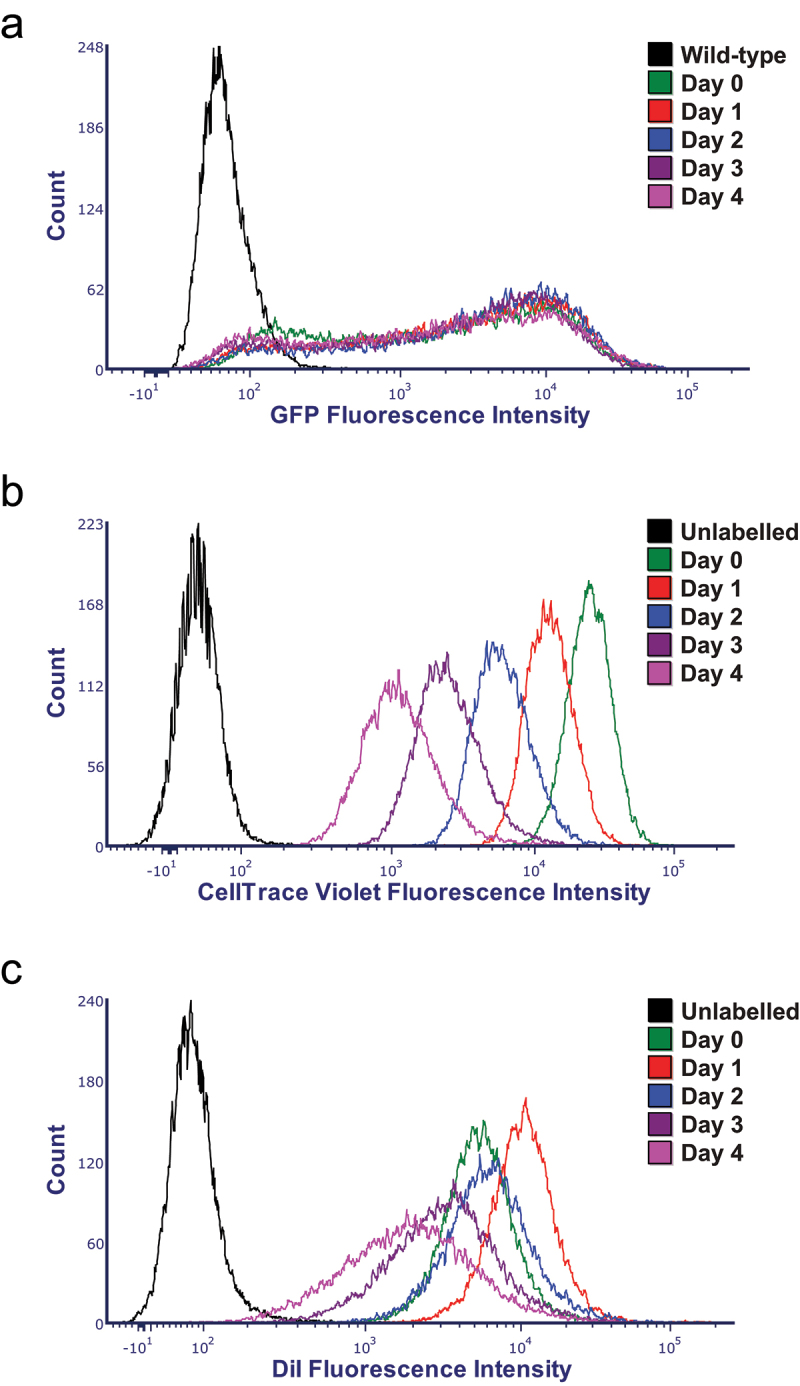
Flow cytometry analysis of GFP-expressing DiI labeled MDA-MB-231 cells. (a–c) Histograms of GFP, CellTrace Violet and DiI fluorescence intensity distribution for the GFP-expressing DiI and CellTrace Violet labeled cells at 0 to 4 days in culture, indicated by green, red, blue, dark and light purple traces, correspondingly. The black trace in (A) corresponds to the histogram for MDA-MB-231 cells that do not express GFP that were used as a control (labeled as wild-type in the figure). The black trace in (B) and (C) corresponds to histograms for DiI-unlabeled GFP-expressing MDA-MB-231 cells used as a control.

For flow cytometry experiments GFP expressing MDA-MB-231 cells were labeled with DiI and CellTrace Violet to track cell proliferation. The cells were split into five equal groups. One group was immediately analyzed with flow cytometry to determine the population of GFP-expressing cells that were also labeled with DiI on Day 0 of the five-day experiment. The other four groups of cells were plated into four different flasks and cultured for one to four days, respectively. Each day, cells from one of the four flasks were collected and analyzed with flow cytometry to determine how the population of the DiI labeled cells changed relative to what was observed on Day 0. Flow cytometry analysis indicated that the GFP intensity for the analyzed viable cells stayed unchanged throughout the experiment and, also, the population of cells expressing GFP remained stable (Figure 6a and Table 1). Flow cytometry analysis using CellTrace Violet indicated that the cells were dividing consistently at about the same rate throughout the experiment (Figure 6b). Flow cytometry analysis using DiI indicated that the DiI labeling on Day 0 was incomplete with about 80% of cells labeled with DiI (Table 1). The population of DiI labeled cells increased on Day 1 of the experiment, followed by a continuous decrease in the next three days of the experiment (Figure 6c). These results suggest that on Day 1 of the experiment DiI was transferred from the labeled cells to the cells that did not get labeled on Day 0. The subsequent decrease in the population of the DiI labeled cells could be due to the uneven transfer of the DiI during the cell division, with some of the daughter cells potentially missing DiI labeling entirely, and retention of DiI in non-viable cells that are excluded for the flow cytometry analysis. The spread of the DiI intensity histograms steadily increased over the duration of the experiment, further supporting the possibility of an uneven transfer of DiI during the cell division that would create subpopulations with different levels of DiI labeling. In summary, our flow cytometry experiments indicate that the DiI labeling levels are not stable and, therefore, do not accurately reflect the number of cancer cells. In contrast the GFP fluorescence remains relatively stable and, therefore, is a better marker for studying the tumor progression for cells that can be modified to express GFP.

**Table 1.** Summary of the flow cytometry analysis for the populations of GFP fluorescent, DiI- and CellTrace Violet labeled cells on the indicated day in cell culture.

Day of the experiment	Cells labeled with CellTrace Violet (%)	Cells labeled with DiI (%)	Cells labeled with GFP (%)	GFP fluorescent cells that are also labeled with DiI (%)
Day 0	99.71	80.12	70.82	62.77
Day 1	84.19	97.89	78.01	71.03
Day 2	18.82	82.42	80.79	72.32
Day 3	1.85	45.36	75.36	50.23
Day 4	0.39	25	71.37	30.41

## Discussion

In this study we investigated the differences between analyzing zebrafish xenografts of GFP-expressing MDA-MB-231 cells with DiI labeling and GFP fluorescence used as means to trace grafted tumor cells. Our results indicate that the xenograft analyses using DiI labeling overestimates the increase in tumor area as compared to the analyses with GFP fluorescence. The overestimation could be due to the transfer of DiI labeling from the cancer cells into the host tissues and retention of the dye in non-viable cells. The transfer of DiI between labeled and unlabeled cells was further supported by the flow cytometry analysis of the cancer cell cultures that indicated an initial increase in the population of DiI labeled cells. The increase was followed by a consistent decline in the population of DiI labeled cells, suggesting that the DiI labeling is either unevenly transferred during the cell division or gets diluted by retention in non-viable cells. Taken together, our results demonstrate the limitations of using DiI for labeling cancer cells and the necessity of verifying the results based on DiI labeling with complementary methods, such as GFP fluorescence.

Both analysis of tumor growth in zebrafish xenografts using DiI labeling and GFP fluorescence indicated a statistically significant increase in the tumor area on Day 3 after the injection (Figures 2b and 3b). Therefore, the two methods yielded qualitatively similar results for the tumor area assessment, however, there was a statistically significant overestimation of the increase when quantified based on the DiI fluorescence. The average percent increase in the tumor area was 34.6 ± 7.2% based on the GFP fluorescence and 52.2 ± 7.9 based on the DiI labeling (Figure 4b). While we used tumor area for assessing tumor growth, as is commonly done for studies using zebrafish xenografts,^27;28^ tumor growth could also be assessed by determining tumor volume using z-stack confocal images. For measuring tumor volume for xenografts over time, the zebrafish need to be immobilized in low-melt agarose with small amount of tricaine, which can negatively affect fish health. In addition, the z-stack image analysis for multiple animals and experimental conditions is more time consuming than the area analysis. Therefore, although tumor volume assessment is more accurate, area analysis is more often used for zebrafish xenografts. The high number of xenografts used in our experiments for calculating the average tumor area (*n* = 37) and comparison of tumor area for the same xenografts imaged on different days of the experiment should minimize the dependence of our conclusions on the cross section used for the area measurements. In addition, since in our study DiI and GFP fluorescence signals are detected from the same cells, the choice of area versus volume for measuring tumor size should have no impact on our conclusions.

The major factors contributing to the significant overestimation of tumor area increase with DiI labeling are most likely transfer of DiI from the labeled MDA-MB-231 cells into zebrafish tissues and also residual DiI labeling of cellular debris as not all cancer cells will survive the injection into a host organism, as suggested by the presence of DiI labeled tumor border regions that are lacking GFP-fluorescence (Figure 3a). These factors have been identified previously as major issues with accurately tracking cells with DiI in vivo^23–26^. Noteworthy, Asokan et al. reported that injection of stably GFP-expressing MDA-MB-231 cells resulted in similar dissemination, extravasation and invasion as that of DiI labeled MDA-MB-231 cells^29^. However, in this study the GFP-expressing and DiI labeled cells were examined separately. In contrast, in our study the GFP-expressing cells were labeled with DiI, affording a direct comparison of the DiI and GFP fluorescence signals. Moreover, quantification of the tumor characteristics monitored with GFP and DiI fluorescence was missing in Asokan et al, making it impossible to exactly determine if there were any differences in the tumor growth assessment based on the GFP and DiI labeling.

Examination of the DiI labeling efficiency with the flow cytometry immediately after the labeling indicated that 20% of the cells were not labeled with DiI (Day 0, Table 1), despite using the optimal labeling conditions. Therefore, complete labeling of MDA-MB-231 with DiI may not be achieved without adversely affecting cell health. The incomplete labeling should not affect our conclusion that the DiI labeling analysis overestimates the tumor growth in zebrafish xenografts as the comparison of the DiI labeling on Day 0 and Day 3 was done for the same fish and, therefore, reflects the relative change in the tumor size regardless of the DiI labeling efficiency. However, the incomplete labeling of MDA-MB-231 cells may be a factor for other studies and should be taken into account during the experimental design.

Interestingly, there was no statistically significant increase in the GFP intensity in xenografts on Day 3 (9.4 ± 5.9%), while the increase in the DiI labeling intensity was statistically significant (35.1 ± 6.7%). Both of these findings seem surprising at first. The GFP-fluorescence would be expected to increase with the increase in the tumor area, while the DiI fluorescence would be expected to stay the same throughout the experiment as the total amount of DiI in the system should be constant. The cancer cell density immediately after the injection into zebrafish embryos is expected to be high and, therefore, the generated GFP and DiI fluorescence signal could potentially saturate the detector. As the cancer cells divide and spread out, the fluorescence signals will also be spread over a larger area and will be within the sensitivity range of the detector. The superficial intensity measurements at the beginning of the experiment could then create misleading results at the end of the experiment. Therefore, using intensity as a measure of cancer cell growth may not be adequate and best to be avoided.

The transfer of DiI from labeled to unlabeled cells observed in the xenograft analysis was in agreement with the results of the flow cytometry experiments that indicated an increase in the number of DiI labeled cells after one day in culture (Figure 6c and Table 1). Noteworthy, after two days in culture the number of DiI labeled cells drastically declined from the peak of ~ 90% after one day in culture to 25% after four days in culture. Most likely the decline is due to the decrease of the DiI labeling intensity as cells divide and also a possible uneven distribution of the DiI during the cell division between daughter cells, as supported by the increased spread of DiI intensity histograms over the course of the experiment (Figure 6c). Indeed, the imaging of DiI labeled cell cultures indicated that DiI is unevenly distributed in the cell membrane (Figure 5). The uneven punctate cell membrane labeling with DiI has been reported before^23;30^ and may reflect a preferential partitioning of DiI-C18 used in our experiments into the “gel” phase of cellular membranes, as reported before.^31;32^ The punctate membrane labeling with DiI could lead to an unequal distribution of DiI in the daughter cells during the cancer cell division. In agreement with this, Figure 4a Day 3 shows invading GFP-expressing cells that lack DiI labeling, suggesting that these daughter cells lost DiI labeling in the process of tumor cell division. This limitation is most pertinent to studies of rapidly proliferating cancer cells and should be inconsequential for tracing cells such as neurons.^21^

The flow cytometry experiments indicated that the intensity of the GFP-expressing cells remained unchanged throughout the experiment (Figure 6a) and the population of GFP expressing cells remained stable over the duration of the experiment (Table 1). The small changes in the population of the GFP-expressing cells over the course of the experiment could be due to the variability in the GFP expression levels in different cells. The GFP detection threshold was set at 0.1% of the fluorescence intensity for the wild-type MDA-MB-231 cells that do not express GFP. Therefore, it is possible that the cells expressing GFP at very low levels may not be detected with the flow cytometry analysis on Day 0, however, their or their daughter cells’ GFP expression may increase on Day 1, contributing to the detected increase in the percent of GFP expressing cells on that day. Overall, the level of GFP fluorescence variability detected with flow cytometry was much smaller than the one detected for DiI labeling, indicating that GFP is a more reliable marker to study cancer cell proliferation than DiI. The GFP signal consistency could be further increased by preselecting the brightest GFP-expressing cells with flow-cytometry for the follow-up xenograft experiments to achieve more uniformly GFP-expressing cell population. Other advantages of using GFP fluorescence for cancer cell tracing include diminished signal from non-viable cells, as dead cells would no longer be producing GFP and absence of fluorescence signal transfer between fluorescent and non-fluorescent cells since GFP is expressed intracellularly and could only leak out of the cells if cell membrane is damaged.^33^

Labeling of cells with DiI and other lipophilic dies is cell type specific, as indicated in the manufacturer’s instructions. DiI transfer from the labeled transplanted cells to host tissues could also occur differently in different animal models. Therefore, the accuracy of the tumor area estimate based on the DiI labeling should be tested for each cell line and animal model. While the focus of this study is on DiI labeling, there are a number of other dyes (reviewed in^34^) that could bypass the hurdles of DiI labeling and are currently underutilized in studies using zebrafish xenografts. For instance, the use of CellTracker dyes, designed for retention inside the cells through several generations, should decrease the transfer of the fluorescence signal from the labeled transplanted cells into the host tissues.

Despite many advantages, using GFP as a cell marker is not without its challenges. Genetically modifying cells for stable expression of GFP is not always an option, especially in the context of PDXs, although, the use of transgenic ubiquitously GFP-expressing animal models should ameliorate this hurdle by providing an option of monitoring patient derived cell growth on the background of GFP-expressing host tissue.^35;36^ The lifespan of the transgenic nude mice ubiquitously expressing GFP is similar to the one of non-transgenic nude mice, indicating that overall GFP is not toxic to animals.^36^ However, there are reports of cytotoxicity of GFP and enhanced sensitivity of GFP expressing cells to anticancer drugs.^37;38^ Finally, proper care should be taken to minimize autofluorescence of tissue that could obscure the interpretation of the results based on the GFP fluorescence.^39^

In summary, our results indicate that tumor growth assessment in the zebrafish xenograft model with GFP fluorescence and DiI labeling qualitatively leads to similar conclusions, however, DiI labeling overestimates tumor growth. Moreover, imaging and flow cytometry experiments indicate that while GFP fluorescence is a reliable marker of cancer cells, DiI labeling changes over time and, therefore, is less reliable marker than GFP. Inconsistencies in protocols and a lack of reproducibility has been identified as a major challenge for the successful use of zebrafish xenograft models.^4^ We hope that our study would increase awareness of limitations of using DiI labeling for cancer cell tracing and would decrease inconsistencies in future studies.

## Materials and methods

### Cell lines and reagents

GFP expressing MDA-MB-231 breast adenocarcinoma cell line was purchased from Cells-Online LLC. The cell line expresses green fluorescent protein as a free cytoplasmic protein and was developed through stable transfection with turboGFP protein. The cells were tested negative for mycoplasma, bacteria, yeast, and fungi by Cells Online LLC. The cells were cultured in Dulbecco’s Modified Eagle Medium (DMEM) supplemented with 10% fetal bovine serum (FBS) at 37°C and 5% CO_2_. All culture media were additionally supplemented with 100 U/mL penicillin, and 100 U/mL streptomycin (Gibco, Carlsbad, CA, USA). Trypsin-free PBS-EDTA was used for cell dissociation and detachment from the tissue culture dishes.

### Zebrafish husbandry and xenografts

All animal procedures were conducted in accordance with NIH guidelines for the care and use of laboratory animals and approved by the Georgetown University Institutional Animal Care and Use Committee.

GFP expressing MDA-MB-231 cells in suspension were labeled with Dil (Thermo Fisher, V22885) according to the manufacturer’s instructions. For the labeling, the cells were incubated with 5 μM DiI for 20 minutes at 37°C as based on our prior experience these are the optimal conditions for MDA-MB-231 cell labeling with DiI. Cells were imaged immediately after the labeling with DiI, to assess the quality of the labeling. Zebrafish embryos were injected with 5 nl of the cell suspension, corresponding to ~ 200 DiI-labeled tumor cells, into the yolk sac at 2-day post fertilization (2dpf), as illustrated in Figure 1. The cells were injected using the General Valve Picospritzer II system. Injected embryos were placed in a 96 well plate with 300 μL fish water and kept in an incubator at 32°C. The embryos were imaged immediately after the injection and 3 days post-injection using Olympus I×71 Inverted Epi-Fluorescent Microscope. Images were analyzed using FIJI – an image processing package based on ImageJ2. The tumor area was selected with the freehand tool in the software and the area and the intensity were used for analysis. Autothreshold with manual adjustment was used. The GFP and DiI fluorescence images were merged in Adobe Photoshop using the “screen” blend mode on unaltered fluorescence microscopy images of the tumors.

### Cell imaging

For the imaging experiments GFP-expressing MDA-MB-231 cells in suspension were labeled with DiI and CellTrace Violet (Thermo Fisher C34571) and seeded into a 8-well glass bottom µ-dish from ibidi GmbH (Germany). For the labeling, MDA-MB-231 cells were incubated with 5 μM DiI and CellTrace Violet for 20 minutes at 37°C. Cells were imaged immediately after the labeling with DiI, to assess the quality of the labeling, and, also, on Day 3 in culture using a Nikon Ti2, W1 SoRa Spinning Disk confocal fluorescence microscope with NIS-ELEMENTS software. The GFP and DiI fluorescence images were z-stacked and merged in FIJI.

### Flow cytometry

DiI and CellTrace Violet (Thermo Fisher C34571) labeled GFP-expressing MDA-MB-231 cells were seeded into four Nunc™ polystyrene cell culture flasks. Each day one flask was washed with PBS, harvested, and analyzed on a BD Fortessa SORP (Becton Dickinson, San Jose CA). Dead cells were excluded from the analysis utilizing Helix NP NIR (Biolegend 425,301). 30000 cells were collected for each sample. Fluorescence signals from the 488 nm laser line excitation were analyzed to detect the GFP and DiI emission at 530 nm and 575 nm respectively, fluorescence signals from 640 nm excitation were analyzed to detect the Helix NIR emission at 660 nm and fluorescence signals from 405 nm excitation were analyzed to detect the CellTrace Violet emission at 450 nm. Autofluorescence of DiI unlabeled cells or wild-type MDA-MB-231 cells that were not genetically modified to express GFP were used as negative controls. The cutoff fluorescence intensity was set so that 99.9% of the control cell autofluorescence. The percentage of positive cells was determined as the percentage of cells that exceeded the cutoff for each of the three parameters (GFP, DiI or CellTrace labeling). Results were analyzed utilizing FCS Express 7 software (DeNovo Software, Pasadena CA).

### Statistical analysis

Comparisons between the GFP and DiI fluorescence signals were performed using the paired-samples Student’s T-test. *P* < .05 was considered statistically significant.

## Data Availability

All data are contained with the article or available on request by contacting the corresponding author: tib5@georgetown.edu.
